# Sole‐ground contact and sitting leg position influence suprahyoid and sternocleidomastoid muscle activity during swallowing of liquids

**DOI:** 10.1002/cre2.216

**Published:** 2019-07-09

**Authors:** Yuta Uesugi, Yoshiaki Ihara, Ken Yuasa, Koji Takahashi

**Affiliations:** ^1^ Division of Oral Rehabilitation Medicine, Department of Special Needs Dentistry, School of Dentistry Showa University Tokyo Japan

**Keywords:** electromyography, posture, swallow

## Abstract

Clinically, the stable sole‐ground contact in the diet is considered as important for achieving safe swallows in the dysphagic patients. However, the effects of varied sole‐ground contacts on swallow‐related muscles activities remain unclear. The aim of this study was to investigate the effects of sole‐ground contacts on the muscle activities during swallow for various materials; 26 healthy adult subjects participated in this study. Three different sole‐ground contact conditions were investigated; sole‐ground contact with knees bent to 90° (KB 90°), sole‐ground contact with knees bent to 135° (KB 135°), and sole‐ground off the floor (Off). Participants swallowed four bolus materials (saliva, 5‐ml water, 10‐ml water, and 5‐ml yogurt) in each sole‐ground contact condition. The muscular activities of the suprahyoid (SH) muscle and the sternocleidomastoid muscle during swallowing were detected and recorded using surface electromyography. The sole‐ground contact pressure was evaluated using the data acquisition system. Duration of SH during 10‐ml water swallow for Off was significantly longer than that for KB 90°. Duration of SH during 5‐ml yogurt swallow for Off was significantly longer than that for KB 90°. Integration of SH during 10‐ml water swallow for Off was significantly greater than that for KB 135°. Integration of SH during 5‐ml yogurt swallow for Off was significantly greater than that for KB 90°. No significant differences were found in peak of SH. Sole‐ground contact conditions had significant effect on swallow‐related muscles activities. The stable sitting positions might be more advantageous for performing effective swallows compared with less stable sitting positions.

## 1 INTRODUCTION

In the treatment of patients with dysphagia, compensatory techniques and rehabilitation strategies to improve dysphagic swallows by altering bolus flow have been widely designed and used (Ohmae et al., 1997; Solazzo et al., 2012). Postural adjustments are one such compensatory technique, improving dysphagic swallows by changing the angle and position of the head and body, thereby resulting in changing the velocity and direction of bolus flow (Ertekin et al., 2001; Park, Seo, Ko, & Park, 2013; Saconato, Chiari, Lederman, & Gonçalves, 2016; Shanahan, Logemann, Rademaker, Pauloski, & Kahrilas, 1993). Both the reclining (Park et al., 2013) and the chin‐tuck position (Saconato et al., 2016) reduce aspiration in dysphagic patients. The clinical benefits of applying postural adjustments singly or in combination are effective in 80–90% of dysphagia patients(Logemann, Rademaker, Pauloski, & Kahrilas, 1994).

Stable sole‐ground contact during eating is important for stability and achieving safe swallows in dysphagic patients (Saitoh & Ueda, 2016). Various sitting postures affect trunk muscle activity (Lee, Lee, & Shin, 2017) and head posture (Kwok, Yip, Cheung, & Yick, 2015). However, the effect of differences in sitting posture on sole‐ground contact, hence on the activity of deglutition muscles, remains unclear (Tables 1 and 2).

**Table 1:** Duration, peak, and integration measured using sEMG for the suprahyoid muscle

Food type		Sole‐ground posture			p value
		Off	KB 90°	KB 135°	
Saliva	Duration (s)	0.70 (0.20)	0.65 (0.19)	0.68 (0.19)	NS
	Peak (μV)	45.44 (16.95)	44.96 (17.31)	43.84 (16.01)	NS
	Integration (sμV)	9.31 (5.64)	9.01 (5.30)	9.05 (5.54)	NS
5‐ml water	Duration (s)	0.76 (0.21)	0.71 (0.21)	0.71 (0.21)	NS
	Peak (μV)	51.10 (17.51)	53.57 (23.37)	48.60 (15.99)	NS
	Integration (sμV)	12.58 (6.73)	12.25 (7.79)	10.91 (6.65)	NS
10‐ml water	Duration (s)	0.82 (0.22)	0.73 (0.22)	0.77 (0.23)	0.003*
	Peak (μV)	52.55 (15.02)	54.69 (17.05)	52.87 (18.57)	NS
	Integration (sμV)	14.58 (6.41)	13.02 (6.83)	12.77 (7.38)	0.025*
5‐ml yogurt	Duration (s)	0.83 (0.22)	0.73 (0.22)	0.78 (0.23)	0.002*
	Peak (μV)	58.30 (16.12)	57.71 (16.71)	57.30 (15.35)	NS
	Integration (sμV)	14.92 (6.39)	12.55 (6.09)	13.72 (7.13)	0.017*

*Note.* Data are provided as means and standard deviations.

Abbreviations: KB, knees bent; NS, not significant; sEMG, surface electromyography.

* *p* < .05.

**Table 2:** Peak and Integration measured from the SCM data

Food type		Sole‐ground condition			p value
		Off	KB 90°	KB 135°	
Saliva	Peak (μV)	10.25 (2.55)	10.89 (2.35)	11.20 (2.85)	0.026*
	Integration (sμV)	1.24 (1.12)	1.11 (0.83)	1.22 (0.91)	NS
5‐ml water	Peak (μV)	10.75 (2.91)	11.73 (3.63)	11.14 (2.85)	NS
	Integration (sμV)	1.61 (1.23)	1.26 (1.18)	1.30 (0.99)	0.037*
10‐ml water	Peak (μV)	11.03 (2.62)	12.79 (6.14)	11.83 (3.12)	NS
	Integration (sμV)	1.72 (1.27)	1.35 (1.43)	1.32 (0.99)	0.049*
5‐ml yogurt	Peak (μV)	11.93 (3.25)	13.58 (6.61)	12.42 (3.15)	NS
	Integration (sμV)	1.83 (1.29)	1.36 (1.78)	1.44 (1.19)	0.015*

*Note.* Data are provided as means and standard deviations.

Abbreviations: KB, knees bent; NS, not significant; SCM, sternocleidomastoid muscle.

* *p* < .05.

Surface electromyography (sEMG) has been used to detect deglutition muscle activities in a number of recent studies. Some of these used sEMG to assess differences in activities of the suprahyoid (SH) and sternocleidomastoid (SCM) muscles during swallowing, and reported differences related to posture and type of food (de Mayo et al., 2005; Inagaki, Miyaoka, Ashida, Ueda, & Yamada, 2007; Miyaoka, Ashida, Kawakami, Tamaki, & Miyaoka, 2010; Steele et al., 2015; Taniguchi, Tsukada, Ootaki, Yamada, & Inoue, 2008; Tsukada, Taniguchi, Ootaki, Yamada, & Inoue, 2009).

In general, bolus accommodation is an autonomic physiological swallow event in response to various food materials. Bolus volume (Miyaoka et al., 2010), food and bolus consistency (Inagaki et al., 2007; Taniguchi et al., 2008; Tsukada et al., 2009), and posture (Inagaki et al., 2007) all influence deglutition muscle activities. However, it is still unclear how sole‐ground contact affects the deglutition muscles during swallowing and which autonomous physiologic adjustments to swallowing occur in response to differences in food materials and volumes. We aimed to investigate the effects of posture on sole‐ground contact and in turn on SH and SCM muscle activities during swallowing in healthy subjects. We also aimed to compare SH and SCM muscle activity elicited during swallowing liquids of different volumes and viscosities (saliva, water, and yogurt) while subjects sat in different postures.

## 2 METHODS

### 2.1 Participants

We recruited 26 healthy adult participants (13 women; mean age 26.4 ± *SD* 3.5 years; range 20–32 years, and 13 men; 28.0 ± 5.7 years; range 21–38 years). None of the participants reported histories or current symptoms of malnutrition, pulmonary disease, neurological disease, musculoskeletal disease, speech or voice disorder, masticating problems, or swallowing disorders. Written informed consent was obtained from each participant. The experiments were approved by the Ethics Committee of the University (2015–11).

### 2.2 Sole‐ground contact modalities and swallowing tasks

**Figure 1:**
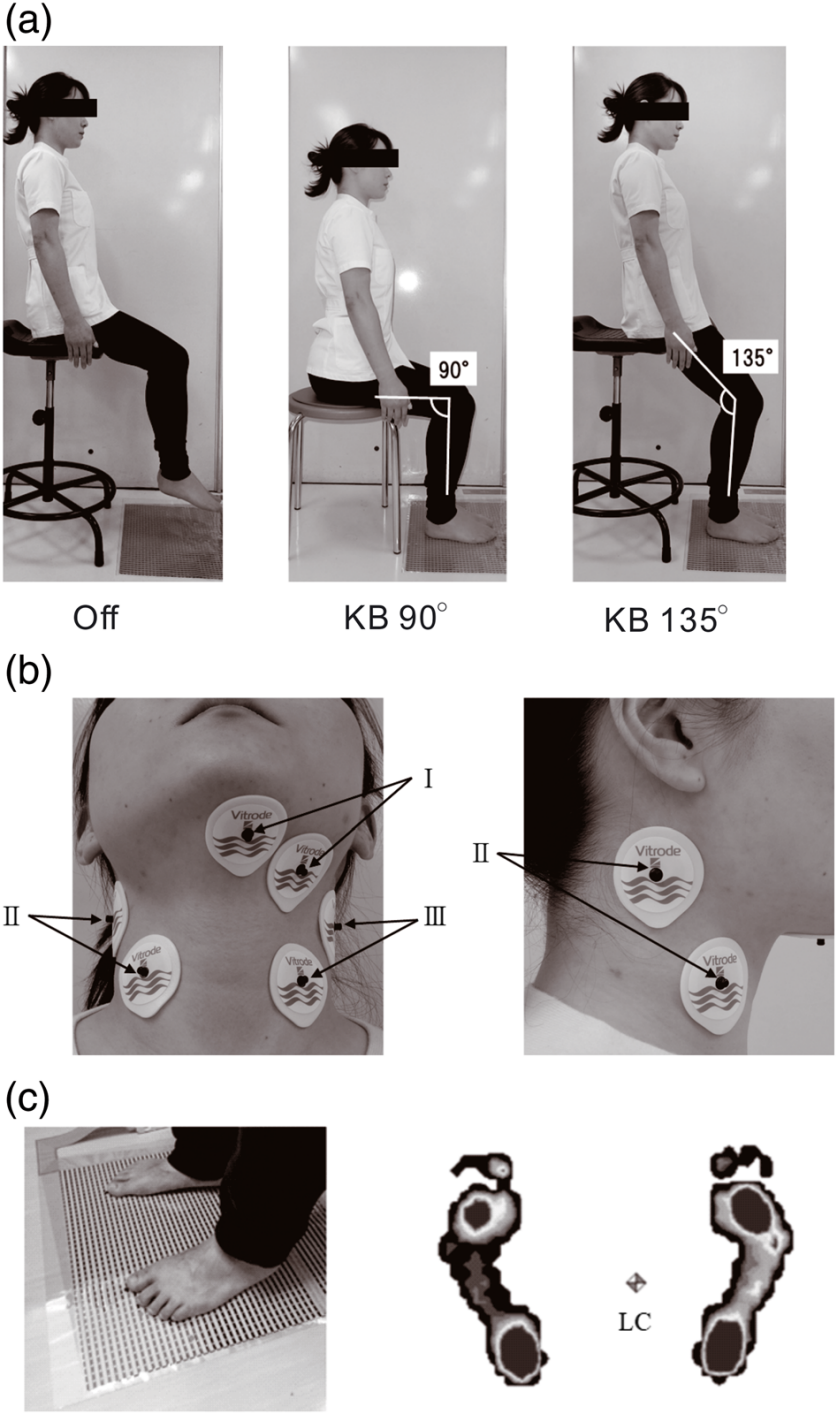
(a) Three different sole‐ground contact postural modalities: left; sole‐ground off the floor (Off), center; sole‐ground contact with knees bent to 90° (KB 90°), and right; sole‐ground contact with knees bent to 135° (KB 135°). (b) Location of electrodes: Bipolar surface electromyography electrodes were located on the skin over left suprahyoid muscle (I) and the skin over both sides of the sternocleidomastoid muscle (II and III). (c) An example of sole‐ground contact pressure measurement during swallowing: left, loading the sole‐ground contact pressure on the sensor sheet as naturally as possible. Right, the dynamic sole‐ground contact condition image. Square mark load center (LC)

Each participant sat on a backless chair. Three different sole‐ground contact modalities were investigated: feet off the floor (Off), and sole‐ground contact with knees bent at either 90° (KB 90°) or 135° (KB 135°). Seat position for KB 135° was higher than that for KB 90° (Figure 1a). Participants were instructed to load the sole‐ground pressure on the sensor sheet of the Body Pressure Measurement System (BPMS, NITTA, Tokyo, Japan, Figure 1c) as naturally as possible at KB 90° and KB 135°. Participants were assigned to perform one of the three modalities in random order during the swallowing task. Each participant kept their torso in a neutral upright sitting position with both arms relaxed at their sides.

The sole‐ground contact pressure data were acquired and recorded using the data acquisition system (BPMS, NITTA, Tokyo, Japan; Figure 1c). Before collecting the data, calibration of each pressure sensor was performed by the BPMS software program for each participant's body weight. The sole‐ground contact pressure data were recorded by the data acquisition system with a 40‐Hz sampling rate.

The sEMG and sole‐ground contact pressure data were acquired simultaneously during the sequential swallowing task, which included five swallowing attempts for each of four bolus materials: (a) saliva, (b) 5‐ml water, (c) 10‐ml water, and (d) 5‐ml yogurt. Except for saliva, bolus materials were administered orally via a syringe, and each participant was instructed to swallow each bolus in a single swallow. A total of 20 swallows were performed as a sequential swallowing task for each of the three sole‐ground contact modalities. To avoid muscular fatigue and loss of concentration during the 20 repeated single swallowing actions, a 5‐min rest period was taken when the sole‐ground contact modality was changed.

### 2.3 sEMG data acquisition

In our preliminary study, the occurrence of muscle action potential detected using sEMG was almost coincident with the beginning of the pharyngeal swallow assessed from simultaneous videofluorographic examination of swallowing (VFSS) images. The discrepancy between the occurrence of muscle action potential detected using sEMG and the beginning of laryngeal elevation verified from VF images was within one video frame (0.03 s).

Bipolar sEMG electrodes (Vitrode J, Nihon Kohden, Tokyo, Japan) were placed on the skin over the left SH complex (Figure 1b, I) and on the skin over both sides of the SCM (Figure 1b, II and III). Neuromotor activities were detected and recorded using sEMG (PowerLab 4/25, AD Instruments, Sydney, NSW, Australia). The sEMG signals were amplified, band‐pass filtered (low cut: 10 Hz, and high cut: 500 Hz), fully rectified, and integrated. These sEMG signals were digitally converted with a 1‐kHz sampling rate and a 16‐bit conversion.

### 2.4 Data analysis

**Figure 2:**
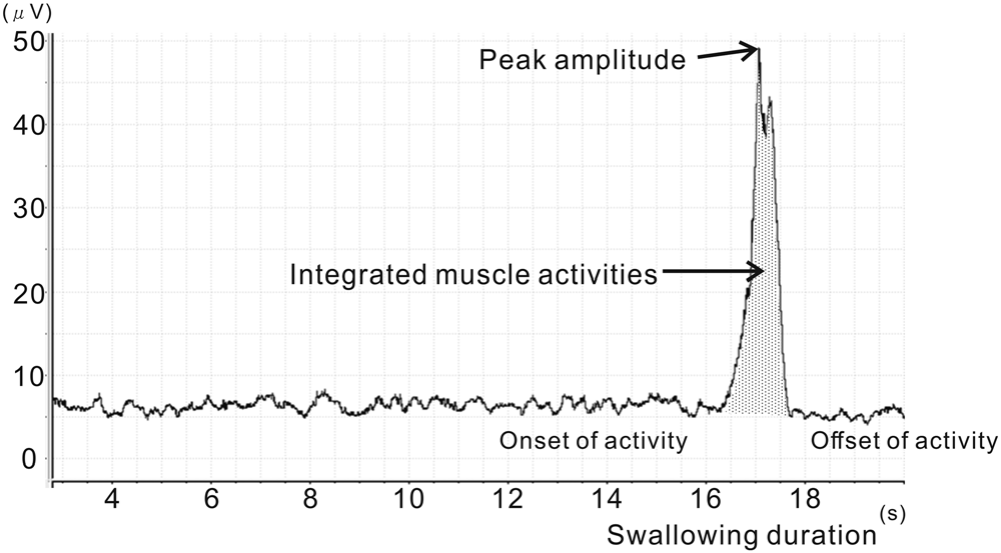
Representative surface electromyography output showing how values for peak amplitude, integration (integrated area under the activity curve), and duration (time from onset to cessation of activity) were obtained

The sEMG data analyses were performed using the data acquisition system (PowerLab 7, ADInstruments, Sydney, NSW, Australia). Swallowing duration (“duration,” seconds: s), peak amplitude (“peak,” μV), and integrated muscle activity (“integration,” sμV) of the SH were measured from the sEMG data. sEMG traces were also evaluated for onset and cessation of activity during swallowing (Figure 2). In accordance with previous studies, onset of activity was identified as the point at which the sEMG signals increased abruptly from baseline. Cessation of activity was identified as the point at which the sEMG signals returned to baseline (Leow, Huckabee, Sharma, & Tooley, 2007). Duration represented the time between the onset and cessation points for each measure. Peak was the highest amplitude (activity) reached over the duration of the action potential, and integration was the area under the activity curve, between onset and cessation. Compared with that of the SH, muscle action potential of the SCM was too low to estimate swallowing duration. Therefore, peak and integration for the SCM were measured over the same time section as duration of the SH (Figure 2). Baseline, duration, and peak data acquired from five repeated swallow attempts were averaged for each bolus material.

### 2.5 Sole‐ground contact pressure analyses

Dynamic sole‐ground contact pressure distribution data were evaluated using a data acquisition system (BPMS, NITTA, Tokyo, Japan; Figure 1c). Total load (kg), contact area (cm^2^), and load center of the sole‐ground contact pressure measurements were derived from the recorded pressure data using the BPMS software program. Total load, contact area, and load center of the sole‐ground contact pressure were measured simultaneously with the peak amplitude of the SH muscle activity. Total load, contact area, and load center of the sole‐ground contact pressure values were obtained during five repeated swallow attempts and averaged for each bolus material.

### 2.6 Statistical analyses

The averaged data obtained for each bolus material were statistically analyzed using SPSS ver. 20.0 (SPSS, Chicago, IL, USA) and JMP pro 13 (SAS Institute Inc., Cary, NC, USA). The Kruskal‐Wallis single factor ANOVA and the Steel‐Dwass test were applied to the sEMG data. The Mann–Whitney U test was used to evaluate the sole‐ground contact pressure data. Unless otherwise stated, values are provided as means ± *SD*, and the significance level was set at *p* ≤ .05.

## 3 RESULTS

### 3.1 Comparisons of SH activity between food types, within each sole‐contact posture

Our sEMG data showed that duration and integration for the SH during 10‐ml water and 5‐ml yogurt swallow, peak for the SCM during saliva swallow, and integration for the SCM during 5‐ml water, 10‐ml water, and 5‐ml yogurt swallow differed significantly among bolus types (Kruskal‐Wallis test; ^*^ *p* < .05).

### 3.2 Comparisons of SH activity between sole‐contact postures, within each food type

**Figure 3:**
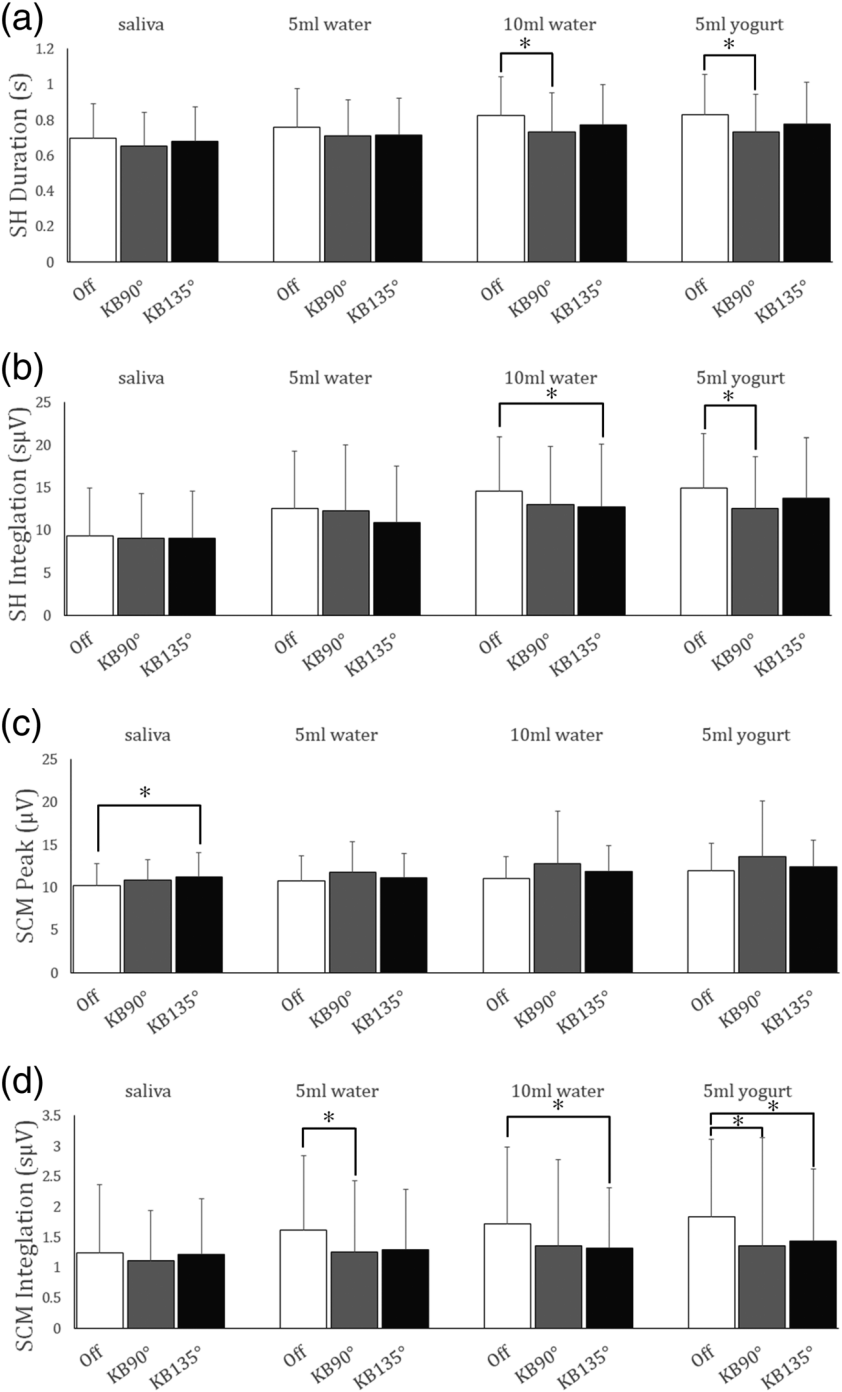
Comparison of surface electromyography data for the suprahyoid (SH) and the sternocleidomastoid (SCM) between different sitting positions and bolus materials. (a) Duration of SH activity, (b) integration for SH activity, (c) peak of SCM activity, and (d) integration for the SCM (*p < .05)

Durations of SH activity during the 10‐ml water and the 5‐ml yogurt swallows were significantly longer for the Off position than for KB 90° (*p* = .001 in both cases, Figure 3a). Integration for the SH during the 10‐ml water swallow was significantly greater for Off than for KB 135° (*p* = .039). Integration for the SH during the 5‐ml yogurt swallow was significantly greater for Off than that for KB 90° (*p* = .018). No significant differences for between postures were found in peak activity for the SH muscle.

### 3.3 Comparisons of SCM activity between sole‐contact postures, within each food type

Peak SCM activity during the saliva swallow was significantly greater for KB 135° than for Off (Figure 3 c; *p* = .020). During the 5‐ml water swallow, integration for the SCM was significantly greater for Off than for KB 90° (*p* = .036; Figure 3d), and during the 10‐ml swallow, it was significantly greater for Off than for KB 135° (*p* = .049; Figure 3d). Integration for the SCM during the 5‐ml yogurt swallow for Off was significantly greater than that for both sole‐contact positions (KB 90°, *p* = .038 and KB 135°, *p* = .027; Figure 3d).

### 3.4 Results of the sole‐ground contact pressure measurement

**Figure 4:**
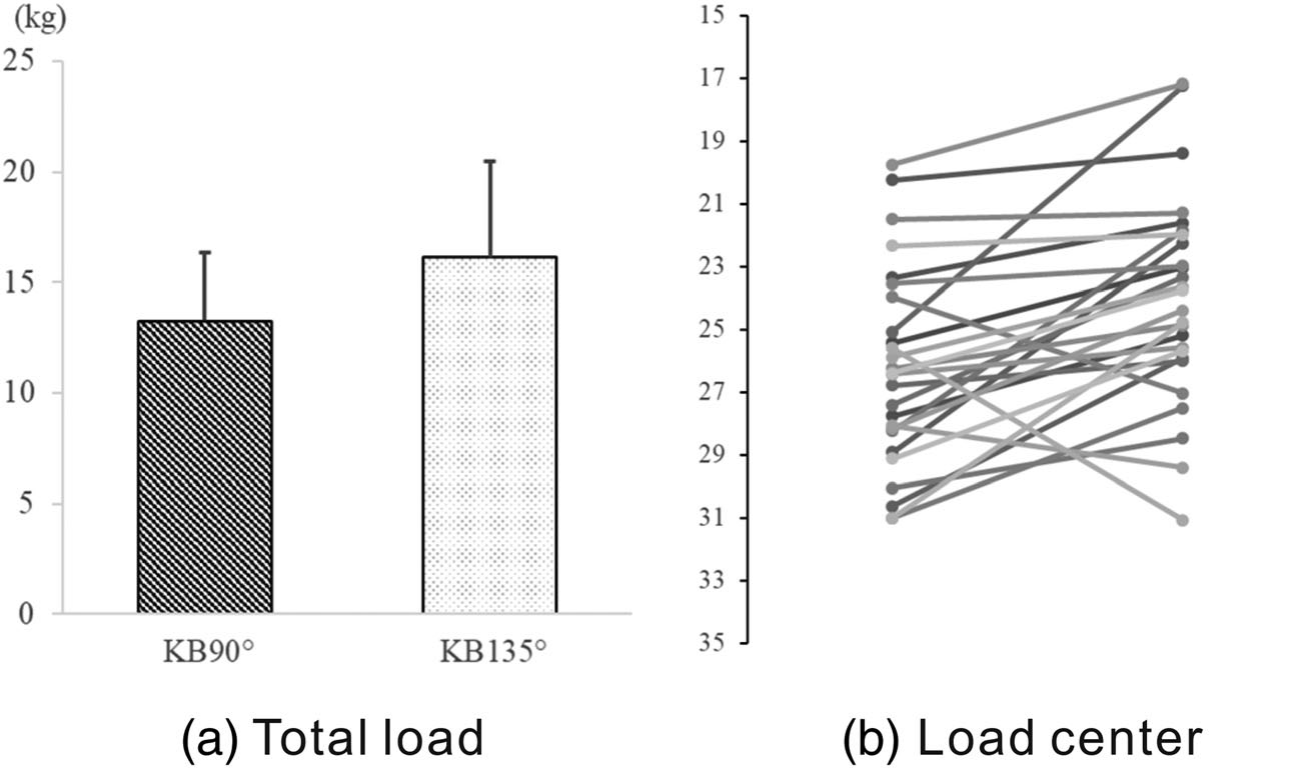
Data acquisition using the Body Pressure Measurement System system. (a) total load and (b) load center (*p < .05)

Total load for all participants seated in the KB 135° position was significantly greater than that during KB 90° (15.80 ± 4.08 kg and 13.57 ± 3.35 kg, respectively, *p* = .030; Figure 4a). Contact area did not indicate any significant difference between KB 90° and KB 135° (Figure 4b). Load center of KB 135° was located anterior (toe side) to that of KB 90° (Mean ± *SD*, KB 135°; 23.88 ± 3.46, KB 90°; 26.33 ± 3.33, *p* = .005; Figure 3c).

## 4 DISCUSSION

Our experiments show that swallowing took longer and required more sustained activity of both the SH when subjects' feet were not in contact with the ground. During repeated swallows of 10 ml of water and 5 ml of yogurt, SH activity duration was significantly higher in the Off posture than when the feet were planted on the ground with the knees at a 90° angle (KB 90°). This effect was less pronounced for saliva and 5 ml of water. Although the SCM showed a smaller activity peak when participants swallowing saliva were seated in the Off posture than in the KB 135°, integral muscle activity for both the SCM and the SH muscles was significantly higher in the Off posture than in postures with sole ground, whether participants were swallowing 10‐ml water or 5‐ml yogurt.

Duration of SH activity during swallowing is known to be related to the properties of food (Igarashi et al., 2010; Inagaki, Miyaoka, Ashida, & Yamada, 2009; van den Engel‐Hoek et al., 2012). van den Engel‐Hoek et al. (2012) reported that viscous liquid elicited significantly longer muscle activity than did saliva and water. In our study, the viscosity of yogurt was higher than that of saliva and water. Bolus volume also influences swallow duration (Kahrilas & Logemann, 1993). Logaman et al. reported that spontaneous saliva swallowing contained approximately 1 ml of liquid (Logeman, 1998). Our study suggests that posture without sole‐ground (Off) leads to longer swallow duration than posture with sole‐ground contact, particularly for high‐volume or high‐viscosity food that requires more effort to swallow.

In this study, SH Peak did not differ significantly among postures or different food types, in agreement with previous work reporting no effect of volume or viscosity of swallowed materials on peak amplitude of muscle activities during swallowing (Shiino et al., 2016). The results of our study suggest that peak amplitude of muscle activity was not affected by posture (sole‐ground contact) or by material swallowed.

The Integral of Off for 10‐ml water and 5‐ml yogurt indicated significant higher number than other posture. Tsukada et al. (2009) reported that there was no significant difference among integrated muscle activities between boluses of 4 ml liquid, 4 ml thin water and thick water. However, we showed that changing of sole‐ground contact conditions influenced integrated muscle activity. This result might suggest that posture without sole‐ground (Off) needs more effort to swallow 10‐ml water and 5‐ml yogurt than other two sole‐ground contact conditions.

The neck muscles influence swallowing effort, with muscle hypertonia causing decreased swallowing efficiency (Ertekin et al., 2002). The result of our study suggested that both increasing in neck muscle activity and absence of sole‐ground contact pressure in the “Off” position might decrease swallowing efficiency in this posture. However, the SCM showed a smaller activity peak when participants swallowing saliva were seated in the Off posture than in the KB 135°. We did not concern about individual salivary flow. It is considered the salivary flow difference was the cause of this results. This requires further study.

The sole‐ground contact pressure measurements indicated significantly greater total load and significantly more forward load center of KB 135° compared with KB 90°. Previous study reported that different degrees of knee flexion influence the trajectory of the center of pressure, peak plantar pressure of sit to stand using various chair heights (Lee & Lee, 2013). Likewise, our results were considered to be due to the higher position of the seat at the KB 90° than that at KB 135°. However, differences of sole‐ground contact condition had a little influence on the muscles activities of SH and SCM during swallow for healthy adults in KB 90° and KB 135°. The fixed height of seat positions across various sole‐ground contact condition will be applied in the future study.

### 4.1 Study limitations

To verify that we were recording muscle activity during the event of swallowing, we conducted a preliminary experiment to verify the time difference between the muscle activities detected by sEMG attached on the skin just above the SH muscle complex, and physiological swallow events, using a combination of sEMG and VFSS. The images were inspected frame by frame to check the timing of the electromyogram and the physiological swallow events. The discrepancy between the occurrence of muscle action potential detected by the sEMG and the beginning of laryngeal elevation verified from VF images was within one video frame (0.03 s). However, this was a pilot study, and we did not use VFSS recording for participants during the study itself. In future, it will be necessary to record swallowing events using concurrent sEMG and VFSS/video endoscopy to investigate which swallowing stage is affected by sole‐ground contact conditions.

It was considered that healthy participants in this study had “autonomous adjustment mechanisms” to keep a stable sitting posture during swallowing of various bolus materials regardless of sole‐ground contact conditions. This might be a reason that sole‐ground contact conditions had no effects on sEMG activities was noted in this study. Future studies investigating the effect of different sole‐ground contact positions on swallowing function in elderly or dysphagic subjects are needed to confirm declining strength of the autonomic adjustment mechanisms of deglutition.

## 5 CONCLUSION

We analyzed muscular activities of the SH and SCM muscles and sole‐ground contact pressure during swallowing of four bolus types in three seated sole‐ground contact postures. Our preliminary study indicated that sole‐ground contact pressure had a significant effect on duration and integration for SH activity and on peak and integration for the SCM muscle. In the management of dysphagic patients during eating or drinking, stable sitting positions with firm evenly distributed sole‐ground contact might achieve greater swallowing effectiveness than less stable sitting positions with the feet off the floor.

## CONFLICT OF INTEREST

The authors declare that they have no conflicts of interest.
